# Expression of Concern: Upregulation of microRNA-497-5p inhibits colorectal cancer cell proliferation and invasion via targeting PTPN3

**DOI:** 10.1042/BSR-20191123_EOC

**Published:** 2020-06-26

**Keywords:** Colorectal cancer (CRC), microRNA-497-5p (miR-497-5p), protein tyrosine phosphatase non-receptor type 3 (PTPN3), proliferation, invasion

The Editorial Office has been made aware of potential issues surrounding the scientific validity of this paper, hence has issued an expression of concern to notify readers whilst the Editorial Office investigates.

